# Circular RNA hsa_circ_0032683 inhibits the progression of hepatocellular carcinoma by sponging microRNA-338-5p

**DOI:** 10.1080/21655979.2021.2024961

**Published:** 2022-01-14

**Authors:** Hao Shen, Haifeng Li, Jiahua Zhou

**Affiliations:** a Department of Hepatic-Biliary-Pancreatic Center, Zhongda Hospital, Medical School, Southeast University, Nanjing, China; b Department of Hepatic-Biliary-Pancreatic Center, Zhongda Hospital, Southeast University, Nanjing, China

**Keywords:** hsa_circ_0032683, miR-338-5p, hepatocellular carcinoma (HCC), RTN4, cell proliferation and apoptosis

## Abstract

Recently, several studies have been conducted on circRNA (circular RNA). circRNA regulates gene expression and plays a vital role in the occurrence and development of various tumors. However, the role and mechanism of hsa_circ_0032683 in hepatocellular carcinoma (HCC) is not studied yet. In GEO (Gene Expression Omnibus) database, hsa_circ_0032683 expression was significantly lower in HCC tissues than in normal liver tissues. In vitro and in vivo functional tests revealed that hsa_circ_0032683 could inhibit HCC cells proliferation and promote their apoptosis. Mechanically, hsa_circ_0032683 primarily exists in the cytoplasm and competes with microRNA-338-5p (miR-338-5p) to regulate reticulon 4(RTN4). Our experiments revealed that hsa_circ_0032683 receded the proliferation ability of HCC via ceRNA (competing endogenous RNAs) mechanism, which provided potential biomarkers and therapeutic targets for HCC patients.

**Abbreviations:** circRNAs: circular RNA; HCC: hepatocellular carcinoma; RTN4: reticulon 4; ceRNA: competing endogenous RNA; GEO: Gene Expression Omnibus; miRNA: microRNA; CSCD: Cancer-specific circRNA database; CRI: Circular RNA Interactome; TCGA: The Cancer Genome Atlas; qRT‐PCR: quantitative real‐time PCR; NEK9:NIMA-related kinase nine; CSMD1: CUB and Sushi multiple domains 1; Tob1: transducer of ERBB2, 1; miR: microRNA; sh: short hairpin; WT: wild type; MUT: mutant

## Graphical abstract

**Figure:**
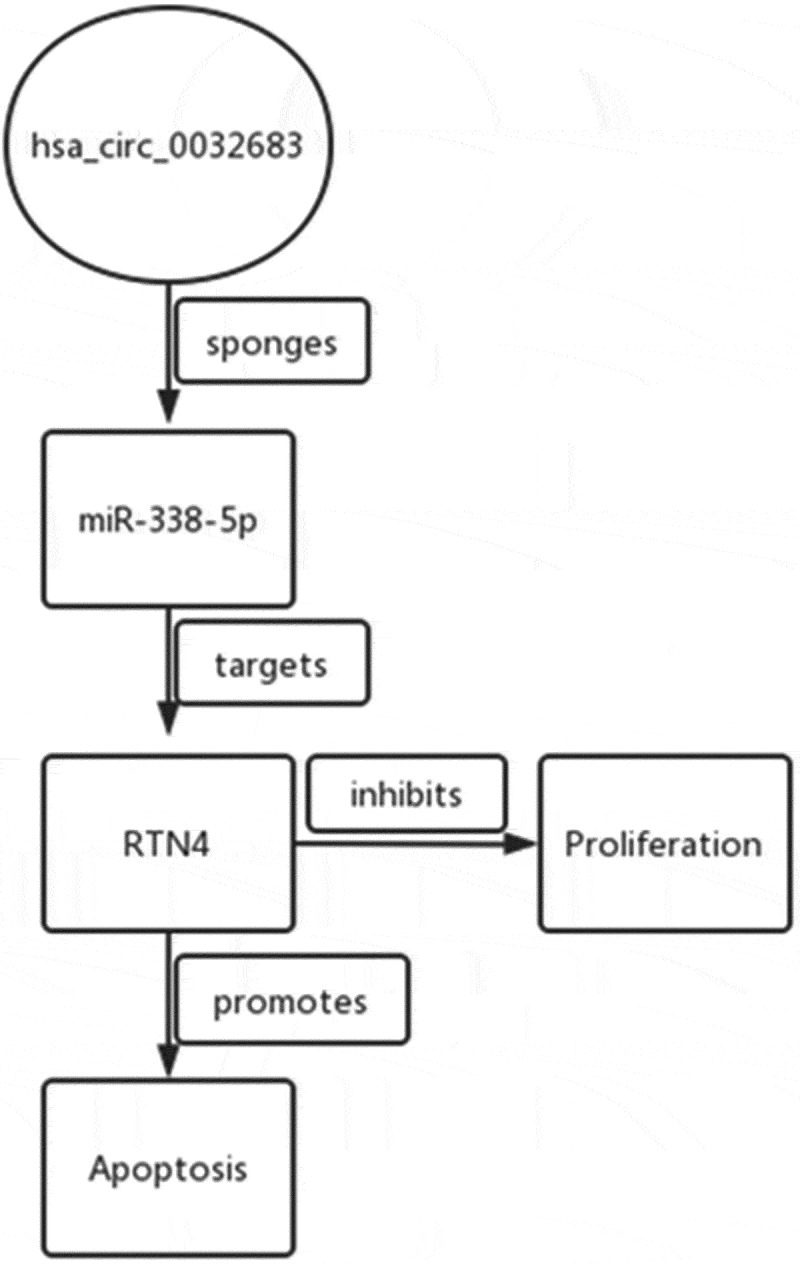

## Introduction

Hepatocellular carcinoma (HCC) is one of the most deadly malignancies worldwide and the second primary cause of tumor-associated death, resulting in more than 700,000 deaths annually [1,2]. Although surgery and liver transplantation have progressed over the last ten years, the 5‐year survival rate of HCC patients is still abysmal due to the high frequency of recurrences and metastases [3–5]. Therefore, discovering novel biomarkers and potential therapeutic targets for HCC is urgently required.

Circular RNAs (circRNAs) were identified in back-splicing precursor mRNA and characterized by a ‘head to tail’ splicing structure [6]. Recently, the expression data of circRNAs could be obtained with great precision using high-throughput sequencing and advanced technology, despite their low levels of expression [7]. Moreover, accruing circRNAs act as ceRNAs (competing endogenous RNAs) in human cancers, such as bladder carcinoma, gastric cancer and hepatocellular carcinoma [8–10]. Studies have revealed that hsa_circ_0041103 could promote the progression of bladder carcinoma through modulating the microRNA(miR)-103a-3p/miR-107- cyclin dependent kinase 6 pathway [8]. circPVT1 may promote gastric cancer cell proliferation by targeting miR-125 [9]. It has been reported in the literature that some circular RNAs are abnormally expressed in the tissues of HCC patients and affect the occurrence and development of HCC. For instance, the hsa_circRNA_0007874 functions as miR-9 sponge to suppress HCC progression [10]. Another example is circRNA_10720, which absorbs microRNAs targeting vimentin, stimulates Epithelial-Mesenchymal Transition (EMT) and promotes HCC tumorigenesis [11]. Due to the relatively stable characteristics of circRNA and its key role in the development of HCC, circRNA can serve as a new diagnostic marker for HCC.

hsa_circ_0032683 is down-regulated in HCC tissues according to the datasets of GSE94508 and GSE97332. The hsa_circ_0032683 is transcribed from chr14:75590748–75590926 (location of the NEK9) (NIMA-related kinase nine genes), approximately 178 bp in length. The hsa_circ_0032683 is a novel circRNA that alleviates gastric cancer’s malignant biological behavior via modulating the miR-409-3p/ microtubule associated protein 7 axis [12]. However, the pathophysiological functions of hsa_circ_0032683 in HCC need to be further explored.

Our research for the first time showed that hsa_circ_0032683 was downregulated in HCC tissues and cell lines. We then investigated the role of hsa_circ_0032683 in tumorigenesis of HCC and provided an insight into the potential molecular mechanisms. The aim of this research was to investigate the regulatory network among hsa_circ_0032683, miR-338-5p and RTN4 (reticulon 4) in regulating the proliferation and apoptosis of HCC. Our research may have some reference value for the diagnosis and prognosis evaluation of HCC patients.

## Materials and methods

### The microarray of circRNAs and bioinformatics processing

The microarray data of circRNAs in HCC of GSE94508 and GSE97332 were obtained from the Gene Expression Omnibus (GEO) database [13]. The raw microarray data were processed using GEO2R. Circbase (http://www.circbase.org/) [14] was used to fetch the biological information of hsa_circ_0032683. The downstream binding sites were searched later. Cancer-specific circRNA database (CSCD) (http://gb.whu.edu.cn/CSCD) and Circular RNA Interactome (CRI) (http://circinteractome.nia.nih.gov/) were used to predict hsa_circ_0032683-binding microRNAs and the binding sites [15,16]. Targetscan (http://www.targetscan.org/), miRTarBase (http://mirtarbase.mbc.nctu.edu.tw/index.html), miRWalk (http://mirwalk.umm.uni-heidelberg.de/), and miRDB (http://mirdb.org/policy.html) were used to explore the downstream mRNAs of miR-338-5p and the possible binding sites [17–20]. Finally, the expression of RTN4 in hepatocellular carcinoma tumour tissues and normal tissues were verified using The Cancer Genome Atlas (TCGA) database [21].

### HCC tissues and cell lines

Forty pairs of HCC tumor tissues and paracancerous tissues were collected from Zhongda Hospital Affiliated to Southeast University since 2018. According to the hepatocellular carcinoma American Joint Committee on Cancer (AJCC) 8th edition staging, the clinicopathological features of the 40 patients with HCC were accurately recorded. All the enrolled patients received a diagnosis of HCC for the first time and did not receive chemotherapy or radiotherapy before the surgery. The study was approved by the Ethics Committee of the Affiliated Zhongda Hospital of Southeast University (Nanjing, China). Written informed consent was obtained from all the patients for tumor sample collection.

In addition, the investigators obtained informed written consent from the subjects. Seven human HCC cell lines (HCCLM3, MHCC97-L, Hep3B, SMMC-7721, Huh7, Bel-7402 and MHCC97-H) and one standard liver cell line (WRL68) were purchased from American Type Culture Collection (ATCC, Manassas, VA, USA). All cells were cultured in a complete medium with Dulbecco’s Modified Eagle Medium, 10% fetal bovine serum (FBS), and 100 ug/ml penicillin and streptomycin (Gibco, NY, USA). The culture was incubated at 37°C with 5% CO_2_.

### Transfection of HCC cells

Lentivirus- hsa_circ_0032683-vector, lentivirus- hsa_circ_0032683, short hairpin RNA (shRNA) targeting RTN4, and its relevant lentivirus were purchased from GenePharma (Shanghai, China). Then, HCCLM3 and MHCC97-L cells were transfected with lentivirus at a multiplicity of infection (MOI) of 10 in the presence of 5 µg/ml puromycin. miR-338-5p mimics and negative control (NC) were purchased from Thermo Fisher Scientific. According to the manufacturer’s instructions, we used Lipofectamine 2000 (Invitrogen) for cell transfection.

### RNA extraction and quantitative real-time PCR

Total RNA was extracted with TRIzol reagent (Invitrogen, Carlsbad, CA, USA) from HCC tissues and cultured cells following the manufacturer’s instructions. For circRNA and mRNA, cDNA was synthesized from total RNA by PrimeScript RT reagent kit (Takara, Dalian, China). qRT-PCR was then performed with a 7500 Real-time PCR System (Applied Biosystems, Carlsbad, CA, USA) according to standard protocols. GAPDH was used as the internal control genes for circRNA and mRNA. Stem-loop primer SYBR Green qRT-PCR were utilized to measure miRNA expression. The expression levels of miRNA were normalized to U6. The primer sequences for hsa_circ_0032683, miR-338-5p, U6, RTN4 and GAPDH were listed in Table 1. 2-ΔΔCt method was used to analyze the relative RNA expression levels. Each reaction was run in triplicate.

**Table 1.** The primers used in this study

Primers	Sequences(5′-3′)
hsa_circ_0032683-Forward	ATGGACAATACCACGCTGCT
hsa_circ_0032683-Reverse	TCTCATTCAAGGCATCACGA
miR-338-5p- Forward	GGGAACAATATCCTGGTGC
miR-338-5p- Reverse	GTGCAGGGTCCGAGGT
U6-Forward	CTCGCTTCGGCAGCACA
U6-Reverse	AACGCTTCACGAATTTGCGT
RTN4-Forward	GCTGTTGGTAGCTGCGGAG
RTN4-Reverse	TCGAGTAGATCCACAGGGTGA
GAPDH-Forward	ACAACTTTGGTATCGTGGAAGG
GAPDH-Reverse	GCCATCACGCCACAGTTTC

### RNase R treatment

The total RNA samples (2 ug) of HCCLM3 and MHCC97-L cells were incubated with RNase R (3 U/μg) (Epicenter Technologies, USA) at room temperature for 30 minutes. Another sample with the same amount of RNA was left untreated. After removal of RNase R, qRT-PCR was used to measure the abundance of hsa_circ_0032683 and NEK9 mRNA respectively.

### RNA fluorescence in situ hybridization (FISH)

The Cy3-labeled hsa_circ_0032683 probes were designed and synthesized by Sangon Biotech (Shanghai, China). HCCLM3 and MHCC97-L cells were grown adherently on round coverslips in 6-well plates, and the cells were permeabilized with 0.5% Triton X-100 and then dehydrated in ethanol. Then we diluted the FISH probe at a concentration of 1:50, and after the denaturation and equilibration process, we added it to the cells overnight. Subsequently, we stained the hybridized cells with DAPI-Antifade for 15 minutes, and then the coverslips were taken out and sealed and placed in the dark for 30 minutes. TCS SP2 AOBS laser confocal microscope (Leica Microsystems, Germany) was finally used to take pictures.

### Cell Count Kit 8 (CCK-8) assay

Cells were seeded in 96-well plates (1000 cells/well in 200 μl complete culture medium) in triplicate. At 0, 24, 48, 72, 96 and 120 h after incubation, 10ul CCK-8 (Dojindo, Tokyo, Japan) was added to each well. After incubation with CCK-8 solution for 2 h at 37°C, the absorbance (450 nm) of each well was measured using a spectrophotometer (Thermo Scientific, Pittsburgh, PA, USA).

### Colony formation assay

Approximately 500 cells were plated in 6-well plates and incubated with the culture medium for 2 weeks. Proliferating colonies (>50 cells/colony) were fixed with methanol for 30 min and stained with crystal violet for 20 min. Finally, images were obtained using a digital camera. All procedures were performed in triplicate.

### 5-Ethynyl-2′-deoxyuridine (EdU) assay

Each group of cells (4 × 104 cells/well) was seeded into 24-well plates with the complete culture medium for 24 h. HCC cells were then incubated with EdU (50 μM) for 2 h at room temperature. Subsequently, each cell group was fixed with 4% formaldehyde for 30 min and permeabilized with 0.5% Triton X-100 for 10 min. Apollo staining and DAPI staining were then performed to identify the EdU-positive cells according to the manufacturer’s instructions. All procedures were performed in triplicate.

### Flow cytometric analysis of apoptosis

FITC Annexin V Apoptosis Detection Kit with PI (BioLegend, San Diego, CA, USA) was applied to measure the apoptotic rate of cells. According to the manufacturers’ instructions, apoptotic cells were resuspended in 100 μl Annexin-binding buffer and stained with 5ul PI and 5ul Annexin V-FITC for 10 min at room temperature in the dark. The samples were finally analyzed by flow cytometry (FACS Calibur; Becton Dickinson, Franklin Lakes, NJ, USA).

### Dual-Luciferase reporter assay

Partial sequences of hsa_circ_0032683 or RTN4 3ʹ -untranslated region (3ʹ-UTR) containing miR-338-5p binding sites were inserted into pmir-GLO vector (Promega, Madison, USA) to generate hsa_circ_0032683 wild type (WT) or RTN4 3ʹ-UTR-WT. We also inserted the mutant sequence into the pmir-GLO vector to generate hsa_circ_0032683 mutant (MUT) or RTN4 3ʹ-UTR-MUT. HCCLM3 cells were transfected with these luciferase reporters and miR-338-5p mimics or NC with Lipofectamine 3000 (Invitrogen). After 24 hours, the transfected cells were obtained to measure the relative luciferase activity by using a Dual-Luciferase Reporter Assay Kit (Promega).

### Mouse xenograft model of HCC

All animal experiments were conducted with approval by the Ethics Committee of Zhongda Hospital Affiliated to Southeast University, following the National Guidelines for the Health Use of Laboratory Animals. Twelve female BALB/c nude mice (aged 4 weeks) were purchased from the Animal Center of Southeast University Medical School. They were randomly divided into the OE group (n =  6) and the negative control (NC) group (n =  6). Mice were housed with ad libitum access to water and food in a temperature- and light-controlled environment. A total of 1 × 10^6^ HCCLM3 cells, stably expressing either hsa_circ_0032683-OE or NC, were injected into the left flank of each mouse. Bidimensional tumor measurements were taken with Vernier callipers every 4 days, and the mice were euthanized after 4 weeks. The volume of the implanted tumor was calculated using the formula: volume =  (width2 × length) / 2.

### Immunohistochemistry (IHC)

The transplanted tumors obtained in the previous experiment were fixed with 4% formalin, and paraffin sections were prepared after embedding in paraffin. Paraffin sections were then incubated overnight at 4°C with primary detection antibodies. Subsequently, the sections were washed three times in PBS and incubated at 37°C for 1 h with anti-rabbit horseradish peroxidase-conjugated secondary antibodies. 3,3-diaminobenzidine solution and hematoxylin were then applied to stain the sections. Finally, we use a microscope to observe the staining results.

### TUNEL assay

After paraffin-embedded sections, the TDT-mediated TUNEL method was used to detect apoptotic cells, and the specific operation was carried out according to the instructions. In simple terms: (1) Routine deparaffinization of sections, xylene immersion 3 times for 10 minutes each, gradient ethanol immersion for 5 minutes; (2) Treatment of tissue with proteinase K working solution at 37°C for 20 minutes; (3) After washing with PBS for 3 times, we added 0.1% Triton to the sections, and incubated at room temperature for 20 minutes; (4) Added the prepared TUNEL reaction solution to the sections, covered with a cover glass, and reacted in a humidified chamber at 37°C for 1 hour; (5) After washing with PBS for 3 times, we added DAPI staining solution to the sections, and incubated at room temperature for 10 minutes in the dark; (6) After the sections were washed and dried, they were mounted with anti-fluorescence quenching mounting tablets. (7) A fluorescence microscope was used to observe the sections and collect images.

The nucleus stained by DAPI is blue under ultraviolet excitation and the positive apoptotic nucleus is green.

### Western blot

Total proteins from HCC cells were extracted with RIPA lysis buffer, separated by SDS-PAGE and then transferred to polyvinylidene difluoride membranes (Merck Millipore, Germany). Polyvinylidene difluoride (PVDF) membranes were blocked in 5% nonfat milk for 2 h and incubated overnight with primary detection antibodies at 4°C. After washing three times in Tris-buffered saline with Tween (TBST), the membranes were incubated with anti-rabbit horseradish peroxidase-conjugated secondary antibody for 1 h at room temperature with gentle rocking. The enhanced chemiluminescence (ECL) method was finally applied to determine protein expression levels. GAPDH served as an internal control.

### Statistical analysis

SPSS22.0 was used to carry out statistical analysis. All of our experiments were repeated three times, and the mean ± SD was used to respond to each group’s parameters. The two-tailed t-tests were used to compare the two groups. The relationship between the expression level of hsa_circ_0032683 and the clinicopathological characteristics of HCC patients was verified using the chi-square test. Log-rank test was used to compare the survival rates between the high hsa_circ_0032683 expression group and low hsa_circ_0032683 expression group by Kaplan-Meier analysis. A *P*-value of <0.05 was considered statistically significant.

## Results

We hypothesized that hsa_circ_0032683 attenuated the progression of HCC through miRNA-338-5p /RTN4 axis. In this research, we confirmed that hsa_circ_0032683 was down-regulated in HCC tissues and cell lines. Moreover, overexpression of hsa_circ_0032683 could inhibit cell proliferation and promote cell apoptosis both in vivo and in vitro. At the mechanism level, we predicted through bioinformatics that hsa_circ_0032683 could function as a miR-338-5p sponge to further regulate the miRNA-targeted gene RTN4 expression to affect HCC proliferation, which was proved by further experiments.

### The expression of hsa_circ_0032683 was reduced in HCC tissues and cell lines

**Figure 1.**
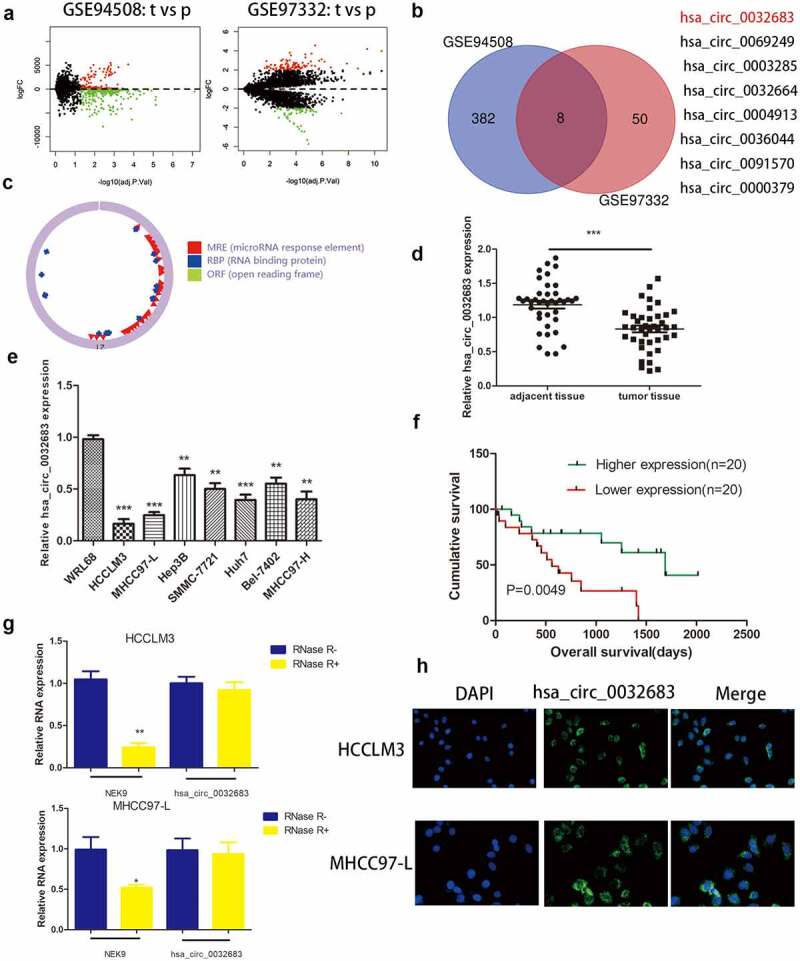
The expression of hsa_circ_0032683 in HCC. (a) Original data were downloaded from GEO database. (b) hsa_circ_0032683 was chosen from 8 downregulated circRNAs. (c)The Circbase and CSCD online tools were used to predict the ring formation characteristics of hsa_circ_0032683. (d) Relative hsa_circ_0032683 expression of 40 HCC patients’ tissues. (e) Relative hsa_circ_0032683 expression of HCC cell lines and WRL68. (f) HCC patients with high expression of hsa_circ_0032683 have a better prognosis. (g) After RNase R treatment, hsa_circ_0032683 expression level and NEK9 mRNA expression. (h) Immunofluorescence localization of hsa_circ_0032683. Data are expressed as the mean ± SD, *P < 0.05, **P < 0.01, ***P < 0.001.

To search the differentially expressed circRNAs in HCC, we discovered two original microarray data with the largest sample size (GSE94508(n = 10), GSE97332(n = 7)) from the GEO dataset (Figure 1(a)). After subsequent R language processing, 16 up-regulated circRNAs and eight down-regulated cercarias were obtained. The filtering threshold was set as follows: LogFoldChange = 2, Adjust P-value = 0.05. We selected 5 circRNAs with the largest expression differences among 16 up-regulated circRNAs and 8 all down-regulated circRNAs and then performed qRT-PCR verification in 15 pairs of HCC and adjacent tissues (Figure S2). We discovered that hsa_circ_0001806 and hsa_circ_0032683 have the most obvious difference through paired-samples T-test. However, it has been proved in the literature that ‘Hsa_circ_0001806 promotes glycolysis and cell progression in hepatocellular carcinoma through miR-125b/ hexokinase 2.’ [22]. Therefore, we studied hsa_circ_0032683 in detail (Figure 1(b)). Using circbase and Chinese Science Citation Database (CSCD) online network prediction tools, we learned that hsa_circ_0032683 is derived from the NEK9 gene and can form a circular structure (Figure 1(c)). 40 HCC patients were randomly selected according to the screening criteria to confirm the prediction. The qRT-PCR verified that the expression level of hsa_circ_0032683 in tumor tissues was significantly lower than that in the corresponding paracancerous tissues (Figure 1(d)).

Similarly, qRT-PCR verified the expression of hsa_circ_0032683 in seven human HCC cell lines (HCCLM3, MHCC97-L, Hep3B, SMMC-7721, Huh7, Bel-7402 and MHCC97-H) was significantly reduced compared with its expression in normal liver cell line WRL68 (Figure 1(e)). To explore hsa_circ_0032683 role in hepatocellular carcinoma and its effect on survival, we performed clinicopathological and survival analyses based on qRT-PCR results. Based on the qRT-PCR results, we classified the patients with a higher expression level than the median value into the high expression group and the patients with a lower expression value than the median value into the low expression group. As shown in Table 2, the low hsa_circ_0032683 expression was associated with tumor size (*P* < 0.01) and Tumor number (*P* < 0.05) of HCC patients. Other clinicopathological measures did not exhibit significant differences. Kaplan-Meier survival analysis revealed that patients with low hsa_circ_0032683 expression achieved worse survival than patients with a high hsa_circ_0032683 expression level (Figure 1(f)). Based on the clinicopathological analysis, we hypothesized that hsa_circ_0032683 might be related to the proliferative capacity of hepatocellular carcinoma. Therefore, we transfected two hepatocellular carcinoma cell lines (HCCLM3 and MHCC97-L) with lentivirus. HCCLM3 and MHCC97-L cell lines are the most different cell lines from normal liver cell line, as validated by previous qRT-PCR analysis. Finally, an RNase R treatment assay was performed to verify the stable presence of hsa_circ_0032683 in HCC cells. As shown in Figure 1(g), NEK9 mRNA expression reduced significantly (*P* < 0.01), but no significant difference was observed in hsa_circ_0032683 mRNA expression in HCCLM3 and MHCC97-L cell lines. The hsa_circ_0032683 was down-regulated and stabilized in HCC tissues and cell lines, and this adjustment might be related to the occurrence and development of hepatocellular carcinoma.

**Table 2.** Expression of hsa_circ_0032683 according to patients’ clinical features

Factors	hsa_circ_0032683 expression		P value
	High	Low	
Gender			
Male	9	10	0.751
Female	11	10	
Age(years)			
≧50	8	11	0.342
<50	12	9	
Tumor size(cm)			
>5	5	14	0.004**
≦5	15	6	
Tumor number			
>1	6	13	0.026*
1	14	7	
Distant metastasis			
Yes	7	9	0.518
No	13	11	

### Subcellular localization of hsa_circ_0032683

Previous results have revealed that hsa_circ_0032683 can be stably expressed in hepatocellular carcinoma cell lines, and the determination of its subcellular localization is a must. As shown in Figure 1(h), hsa_circ_0032683 was primarily present in the cytoplasm of HCCLM3 and MHCC97-L cells. These results provided a basis for us to study the biological function of hsa_circ_0032683.

### Hsa_circ_0032683 overexpression inhibited the proliferation ability of hepatocellular carcinoma in vitro and in vivo

**Figure 2.**
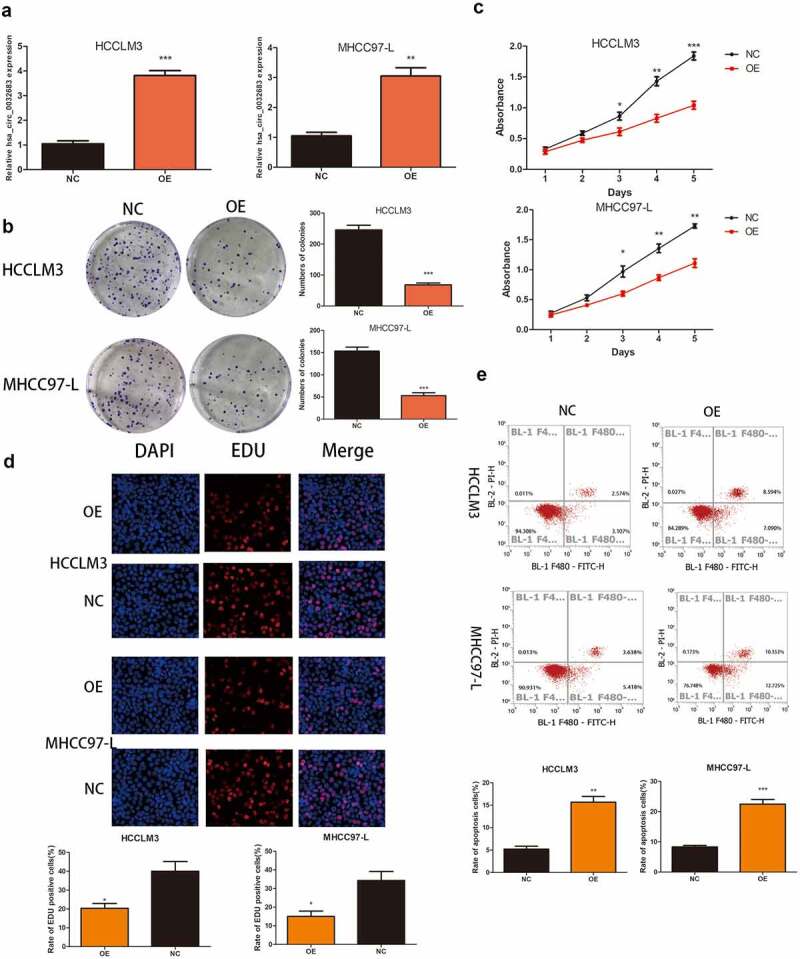
Up-regulation of hsa_circ_0032683 suppressed cell proliferation and promoted apoptosis. (a) The expression levels of hsa_circ_0032683 in HCCLM3 and MHCC97-L cells transfected with OE- hsa_circ_0032683. (b) hsa_circ_0032683 overexpression inhibited colony formation of HCC cells. (c) The proliferation capacities of HCCLM3 and MHCC97-L cells transfected with OE- hsa_circ_0032683 were detected by CCK-8 assays. (d) Up-regulation of hsa_circ_0032683 inhibited cell growth of HCCLM3 and MHCC97-L cells by Edu assay. (e) hsa_circ_0032683 could promoted cell apoptosis by flow cytometry. Data are expressed as the mean ± SD, *P < 0.05, **P < 0.01, ***P < 0.001.

qRT-PCR results indicated the successful construction of the overexpressed cell lines HCCLM3 and MHCC97-L (Figure 2(a)). As shown in Figure 2(b,c), the hsa_circ_0032683 high expression group exhibited lower clone formation and proliferation ability in HCCLM3 andMHCC97-L cell lines than the control group (*P* < 0.001, *P* < 0.01). Similarly, the EDU assay revealed that the cell proliferation rate in the hsa_circ_0032683 overexpression group was remarkably lower than that in the control group (*P* < 0.05) (Figure 2(d)). Flow cytometry analysis indicated that the hsa_circ_0032683 overexpression group exhibited a higher percentage of apoptosis in HCCLM3 and MHCC97-L cell lines (*P* < 0.01) (Figure 2(e)). At the same time, we constructed a murine xenograft model to detect the proliferation promoting effect of hsa_circ_0032683 in vivo. The tumor size, weight, and volume of the hsa_circ_0032683 overexpression group were smaller than those in the control group (Figs. S1A, B, and C). Immunohistochemical staining was applied to determine the expression of cell proliferation markers (Ki-67) in the tumors. TUNEL assay was utilized to confirm the levels of apoptosis in the tumor tissues of hsa_circ_0032683 overexpression and control groups. The hsa_circ_0032683 overexpression group had lower proliferation ability and higher apoptosis rate than the control group (Figs. S1D, and E). These results suggested that the overexpression of hsa_circ_0032683 could suppress the proliferation of HCC cells and promote their apoptosis in vitro and in vivo.

### Hsa_circ_0032683 could sponge miR-338-5p

**Figure 3.**
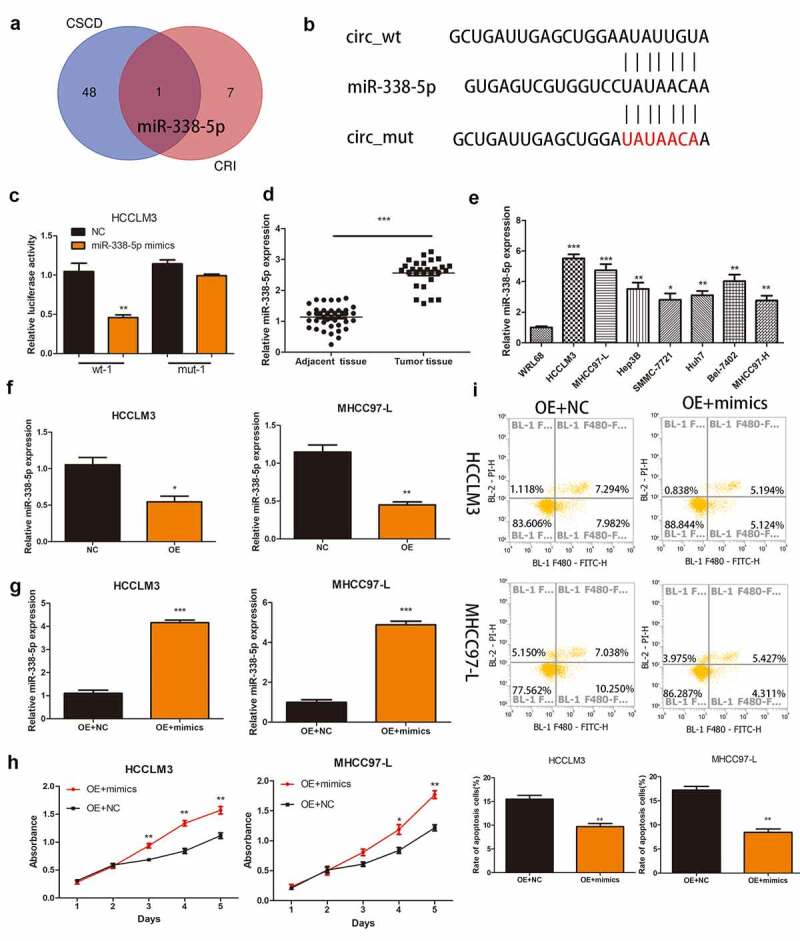
Hsa_circ_0032683 may act as the miR-338-5p sponge. (a) Potential targets were screened out through CSCD and CRI. (b) The schematic diagram of the binding sites between miR-338-5p and hsa_circ_0032683. (c) The relative luciferase activity detected by the dual-luciferase reporter assay. (d) Relative miR-338-5p expression of 40 HCC patients’ tissues. (e) Relative miR-338-5p expression of HCC cell lines and WRL68. (f) miR-338-5p expression level in HCCLM3 and MHCC97-L cells after transfected with OE- hsa_circ_0032683 or NC. (g) miR-338-5p expression level in transfected HCCLM3 and MHCC97-L cells. (H) Overexpression of miR-338-5p accelerated cell growth by CCK-8 assay. (I) Overexpression of miR-338-5p inhibited cell apoptosis by flow cytometry. Data are expressed as the mean ± SD, *P < 0.05, **P < 0.01, ***P < 0.001.

To investigate the mechanism of action of hsa_circ_0032683 in hepatocellular carcinoma, we predicted the hsa_circ_0032683-binding miRNAs using the CSCD and CRI. After considering the intersection of the two databases, we discovered miR-338-5p that binds with hsa_circ_0032683 (Figure 3(a)). The potential binding sites between hsa_circ_0032683 and miR-338-5p are shown in Figure 3(b). Luciferase assays were subsequently carried out to confirm whether hsa_circ_0032683 can combine with the miR-338-5p. Construction of hsa_circ_0032683 wild-type sequence (wt-1) and hsa_circ_0032683 mutant sequence (mut-1) plasmid revealed that miR-338-5p mimics mitigated hsa_circ_0032683-regulated luciferase activity in wt-1 compared with mut-1 in HCCLM3 cells (Figure 3(c))). qRT-PCR was used to measure the expression of miR-338-5p in HCC tissues and cell lines. As is shown in Figure 3(d,e), miR-338-5p were more highly expressed in HCC tissues and cell lines than normal tissues and WRL68 cell lines. However, the expression of miR-338-5p was significantly decreased in the hsa_circ_0032683 high expression group than the control group (Figure 3(f)). Finally, the CCK-8 assay detected the effect of miR-338-5p on HCC cell function. We first transfected miR-338-5p and control lentivirus into hsa_circ_0032683 overexpressed cells (HCCLM3 and MHCC97-L) and verified the transfection efficiency using qRT-PCR (Figure 3(g)). The miRNA-338-5p overexpression group exhibited higher proliferation ability and a lower percentage of apoptosis in HCCLM3 and MHCC97-L cell lines (Figure 3(h,i)).

### RTN4 might be a gene target of miRNA-338-5p in HCC cells

**Figure 4.**
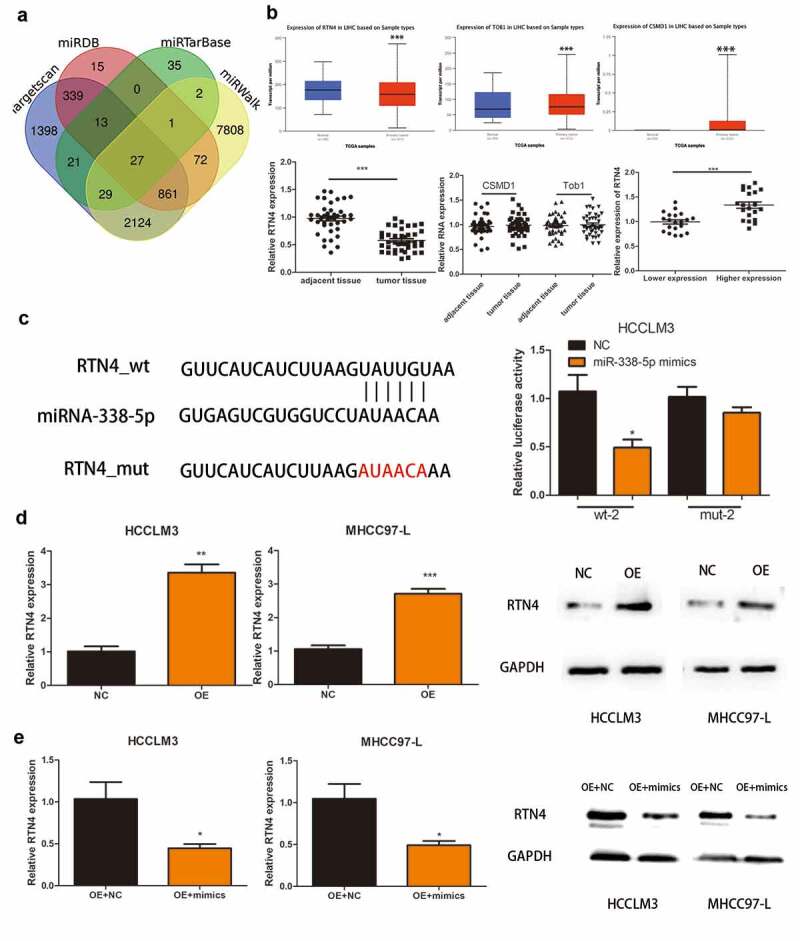
RTN4 may be a potential target of miR-338-5p. (a) Potential targets were screened out through miRTarBase, miRWalk, Targetscan and miRDB. (b) The expression of CSMD1, RTN4 and Tob1 were verified through TCGA databases and 40 pairs of HCC tissues collected. The last picture shows the relative expression of RTN4 in HCC tissues of hsa_circ_0032683 high expression group and hsa_circ_0032683 low expression group. (c) The relative luciferase activity detected by the dual-luciferase reporter assay. (d) RTN4 mRNA and protein expression in HCCLM3 and MHCC97-L cells transfected with OE- hsa_circ_0032683. (e) RTN4 mRNA and protein expression in HCCLM3 and MHCC97-L cells transfected with OE- miR-338-5p. Data are expressed as the mean ± SD, *P < 0.05, **P < 0.01, ***P < 0.001.

**Figure 5.**
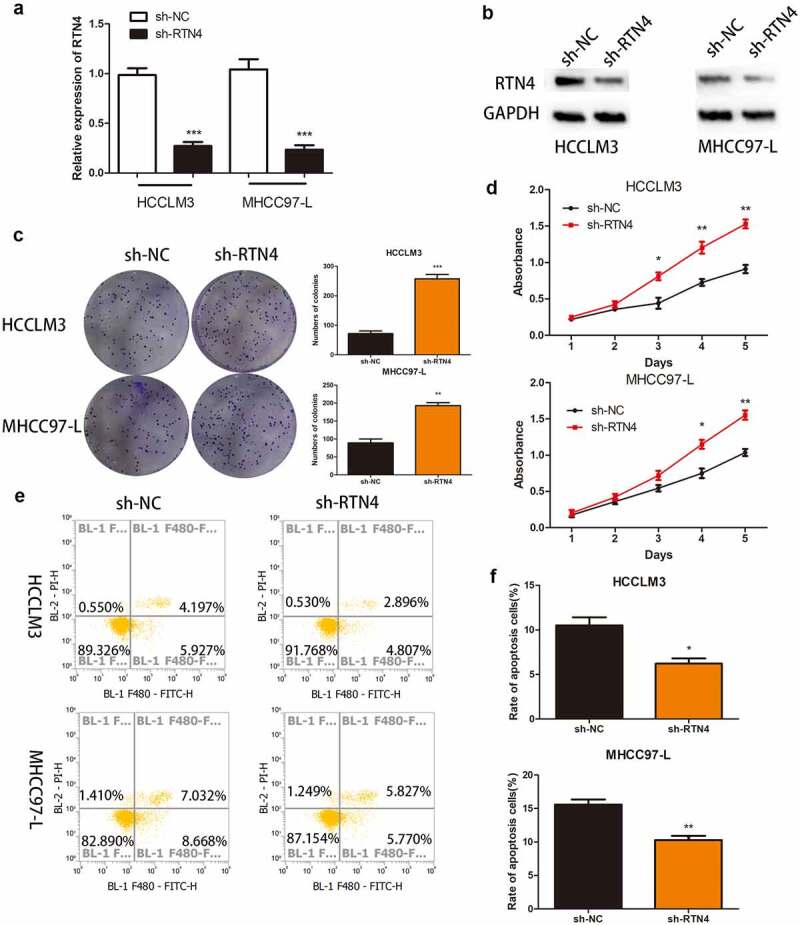
The effect of RTN4 on proliferation and apoptosis in hsa_circ_0032683 overexpressed HCC cells. (a) The level of RTN4 mRNA was measured by qRT-PCR in HCCLM3 and MHCC97-L cells after treating with shRNA against RTN4. (b) Western blotting was performed to measure the RTN4 protein level. (c and d) Knockdown of RTN4 in hsa_circ_0032683 overexpressed HCC cells accelerated colony formation and growth of HCC cells. (e and f) Knockdown of RTN4 in hsa_circ_0032683 overexpressed HCC cells inhibited cell apoptosis of HCCLM3 and MHCC97-L cells by flow cytometry. Data are expressed as the mean ± SD, *P < 0.05, **P < 0.01, ***P < 0.001.

We identified 27 genes as the potential target of miRNA-338-5p using online tools, namely Targetscan, miRTarBase, miRWalk, and miRDB (Figure 4(a)). Previous experiments confirmed that miRNA-338-5p promoted the proliferation of HCC, so the selected downstream target gene must possess tumor growth and proliferation inhibition properties. Among the 27 genes, only CUB and Sushi multiple domains 1 (CSMD1), RTN4, and transducer of ERBB2, 1 (Tob1) were confirmed as tumor suppressor genes. Then, we used The Cancer Genome Atlas (TCGA) database to verify the expression levels of these three genes in HCC patients, and the results revealed that RTN4 was down-regulated in HCC patients (*P* < 0.001), whereas CSMD1 and Tob1 were up-regulated in HCC patients (*P* < 0.001) (Figure 4(b)). Subsequently, we verified this phenomenon in 40 pairs of HCC tissues collected, and the results revealed that RTN4 exhibited low expression in tumor tissues (*P* < 0.001). No significant difference was observed in the expression levels of CSMD1 and Tob1 between tumor tissues and paired par cancer tissues (Figure 4(b)). In addition, qRT-PCR results showed that the expression level of RTN4 in HCC tissues of the hsa_circ_0032683 high expression group was higher than that of the hsa_circ_0032683 low expression group (*P* < 0.001) (Figure 4(b)). Therefore, we targeted the downstream target gene of miRNA-338-5p on RTN4 and selected it for subsequent studies. Dual-luciferase reporter gene assay revealed that miR-338-5p mimics mitigated RTN4-regulated luciferase activity in RTN4 wild-type sequence (wt-2) compared with a RTN4 mutant (mut-2) in HCCLM3 cells (*P* < 0.05, Figure 4(c)). The mRNA and protein expression of RTN4 was up-regulated in the hsa_circ_0032683 overexpression group compared with the control group (*P* < 0.01) (Figure 4(d)). However, the mRNA and protein expression level of RTN4 was remarkably down-regulated in HCCLM3 and MHCC97-L cells when miR-338-5p was overexpressed (*P* < 0.05) (Figure 4(e)). RTN4 could inhibit the proliferation and promote apoptosis of HCC cells [23]. However, no relevant literature has shown the internal relationship between hsa_circ_0032683 and RTN4. We cotransfected the lentivirus- hsa_circ_0032683 and shRNA targeting RTN4 (sh-RTN4) into HCC cells to verify the effect of RTN4 on proliferation and apoptosis in hsa_circ_0032683 overexpressed HCC cells. Depletion of RTN4 was verified by qRT-PCR and Western blot analysis (Figure 5(a,b)). As shown in Figure 5(c,d), knockdown of RTN4 could reverse the inhibitory effect of hsa_circ_0032683 overexpression on HCCLM3 and MHCC97-L proliferation (P < 0.01). In addition, among HCCLM3 and MHCC97-L cells overexpressed by hsa_circ_0032683, the RTN4 knockdown group could significantly reduce the apoptosis rate of HCC cells compared with the control group (p < 0.05) (Figure 5(e,f)). In summary, miR-338-5p might target the RTN4 mRNA to enhance the proliferation ability of HCC, and hsa_circ_0032683 might regulate RTN4 by sponging miR-338-5p.

## Discussion

Although several treatments for HCC exists, including partial liver resection, liver transplantation, and molecular targeted drugs, the five-year survival rate for HCC patients is still not ideal [24,25]. Several studies have illustrated that the abnormal expressions of circRNA play a crucial regulatory role in HCC [10,26,27]. Therefore, we studied circRNA to discover possible therapeutic targets for HCC. The current study primarily screened circRNAs with low expression in HCC tissues using the GEO database and discovered hsa_circ_0032683, which previous studies have not reported. Through clinical correlation analysis and prognosis analysis, we discovered that the expression level of hsa_circ_0032683 was correlated with the size and number of HCC. Patients with high expression of hsa_circ_0032683 were associated with a better prognosis. Previous studies have revealed that abnormal circRNA expression could lead to changes in the biological behavior of HCC [10]. Therefore, we designed in vitro functional tests and in vivo tumor-forming models in nude mice to verify the effects of hsa_circ_0032683 on HCC proliferation and apoptosis.

Further research revealed that hsa_circ_0032683 could enhance the expression of RTN4 in HCC by regulating miR-338-5p. Many studies have demonstrated that miR-338-5p plays a crucial role in the occurrence and development of many tumors. For instance, miR-338-5p promotes the proliferation and metastasis of melanoma cells by targeting CD82 molecule [28]. MiR-338-5p promotes the invasion and metastasis of glioma cells by targeting teashirt zinc finger homeobox 3 and matrix metallopeptidase 2 [29]. A bioinformatics article has systematically described the role of miR-338-5p in HCC, and the results revealed that miR-338-5p might serve as a promising diagnostic marker for HCC [30]. Additionally, miR-338-5p could affect HCC development by targeting specific downstream genes and pathways. However, this paper does not carry out experimental verification. In addition, some studies have demonstrated that Long non-coding RNA (lncRNA) and circRNA could regulate the expression of miR-338-5p in tumors. For example, lncRNA mBLN1-AS1 inhibited the growth and proliferation of retinoblastoma by targeting the miR-338-5p-Wnt/β-catenin signaling pathway [31]. Circular RNA circ_0001649 inhibited the proliferation of non-small cell lung cancer by sponging miR-331-3p and miR-338-5p [32]. However, the sponge adsorption of miR-338-5p by hsa_circ_0032683 has not been reported. For the first time, miR-338-5p was verified to be up-regulated in HCC tissues and cell lines. We performed a dual-luciferase reporter gene assay based on bioinformatics prediction to confirm that hsa_circ_0032683 and miR-338-5p exhibited direct base complementary pairing. Finally, we concluded that hsa_circ_0032683 functions as a tumor suppressor gene by down-regulating miR-338-5p.

We used online software to predict the downstream target genes of miR-338-5p and focused on RTN4. RTN4 was located on chromosome 2p12-14 and implicated in the genesis and progression of many tumors [33,34]. RTN4 was involved in many cell activities, such as cell proliferation, differentiation, and apoptosis, and malfunctioning of these mechanisms could lead to cancer development [23]. Studies have revealed that RTN4 is down-regulated in HCC tissues and cell lines, and high expression of RTN4 might inhibit the growth and proliferation of HCC and promote tumor apoptosis [23]. In our study, we verified the low expression of RTN4 in HCC tissues using the TCGA database and clinical specimens collected by ourselves. Dual-luciferase reporter assay revealed a binding phenomenon between miR-338-5p and RTN4 mRNA. The results revealed that RTN4 was positively correlated with the expression level of hsa_circ_0032683, whereas RTN4 was negatively correlated with miR-338-5p mRNA expression level. Overall, hsa_circ_0032683 up-regulated RTN4 expression by acting as a miRNA sponge to adsorb miR-338-5p to attenuate the proliferation ability of HCC cells and promote apoptosis.

miR-338-5p has been reported to inhibit malignant biological behaviors of HCC cells by targeting ATP binding cassette subfamily B member 1 and epidermal growth factor receptor [35]. However, this is not inconsistent with our research results because miRNA regulates tumor cell proliferation and apoptosis in many aspects. Our research assists in the construction of the HCC regulatory network. With the comprehensive construction of the regulatory network, the mechanism of HCC occurrence and development will be gradually revealed. However, some limitations exist in our research. Tumors with the same histological characteristics might be remarkably different at the genetic level, and miR-338-5p might perform various functions in other HCC tumors. Therefore, we must select more hepatocellular carcinoma cell lines for further exploration.

In recent years, there have been many studies on the role of circRNAs in HCC [10,36]. The current study is the first to discover the regulatory axis hsa_circ_0032683-miR-338-5p-RTN4, which might become a therapeutic target for HCC patients. Moreover, our research is also one of the few to further knock down target genes in cells overexpressing circRNA to verify the role of hsa_circ_0032683/miR-338-5p/RTN4 axis in HCC. This makes our conclusion more convincing. In future studies, we shall combine the results of the current experiment with the existing targeted drugs for HCC, such as sorafenib, and apply them to HCC patients to help them obtain the chance of surgery and improve their prognosis.

## Conclusion

Our results revealed that hsa_circ_0032683 up-regulated RTN4 expression by acting as a miRNA sponge to adsorb miR-338-5p to attenuate the proliferation ability of HCC cells and promote apoptosis. Therefore, the hsa_circ_0032683-miR-338-5p-RTN4 axis could be a potential target for HCC treatments.

## Supplementary Material

Supplemental Material

## Data Availability

Publicly available datasets were analyzed in this study. This data can be found here: https://portal.gdc.cancer.gov. The two GEO datasets referenced are GSE94508 and GSE97332

## Highlights

- hsa_circ_0032683 was downregulated in HCC tissues and cell lines.
- hsa_circ_0032683 receded the proliferation ability of HCC.
- hsa_circ_0032683 modulates RTN4 expression though targeting miR-338-5p.
- The role of hsa_circ_0032683/miR-338-5p/RTN4 axis in HCC.
