# Supplementary figures and images for: Circular RNA hsa_circ_0032683 inhibits the progression of hepatocellular carcinoma by sponging microRNA-338-5p
Source: Bioengineered. 2022 Jan 14;13(2):2321–35. doi: 10.1080/21655979.2021.2024961 (PMC8974012; doi:10.1080/21655979.2021.2024961)

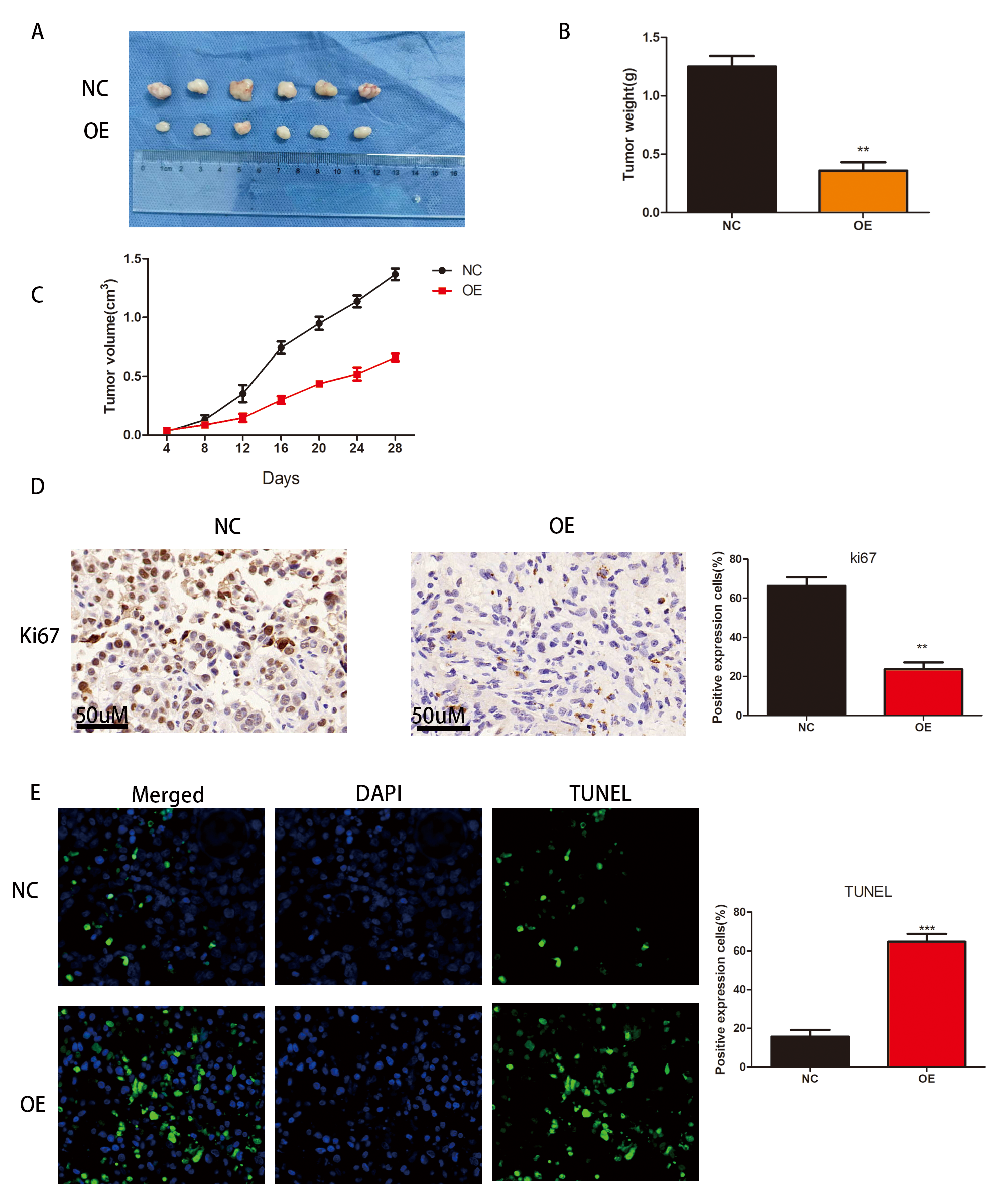

Supplement: Supplemental Material [file KBIE_A_2024961_SM8694.zip › supplementary/Figs1.tif]

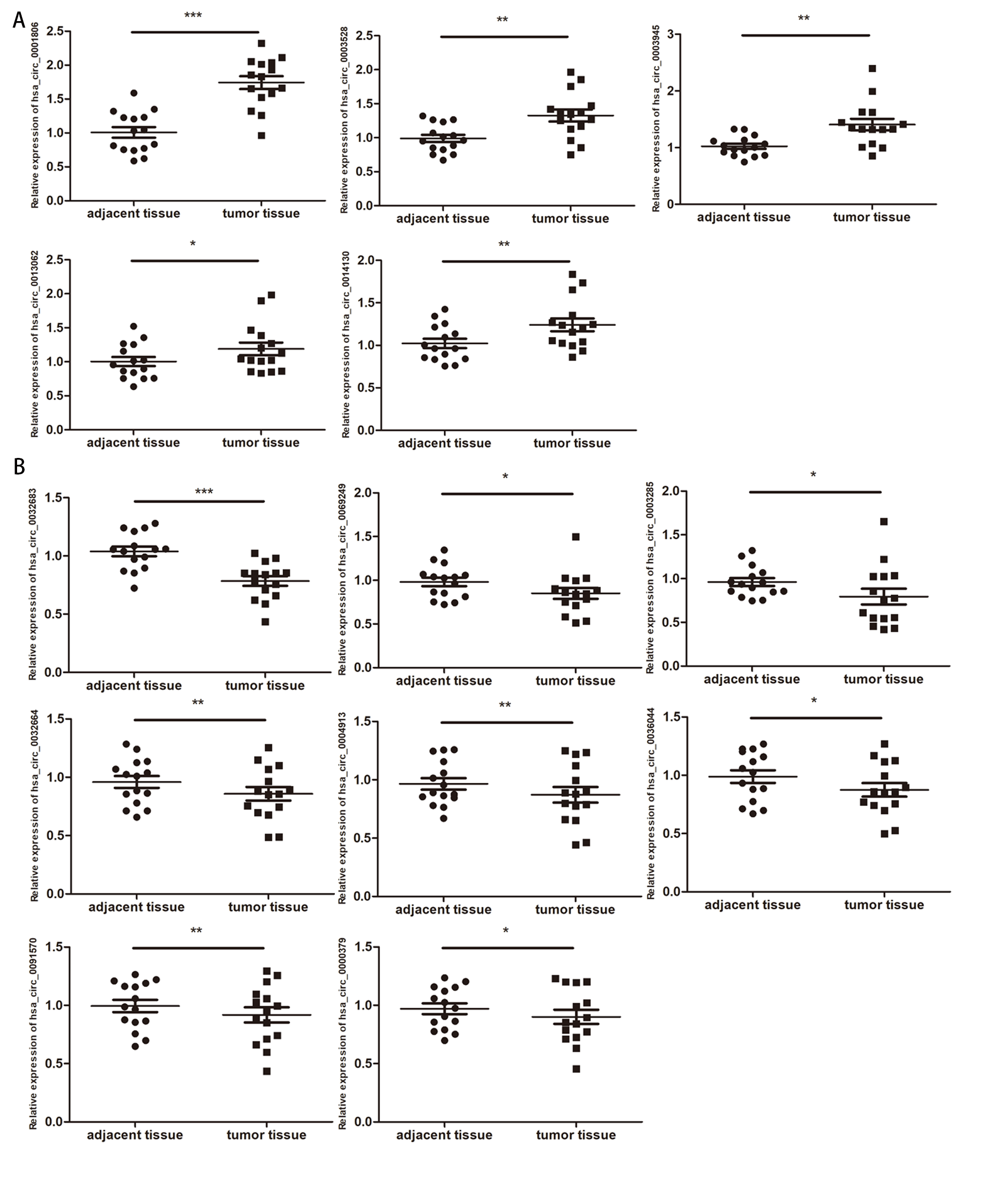

Supplement: Supplemental Material [file KBIE_A_2024961_SM8694.zip › supplementary/Figs2.tif]

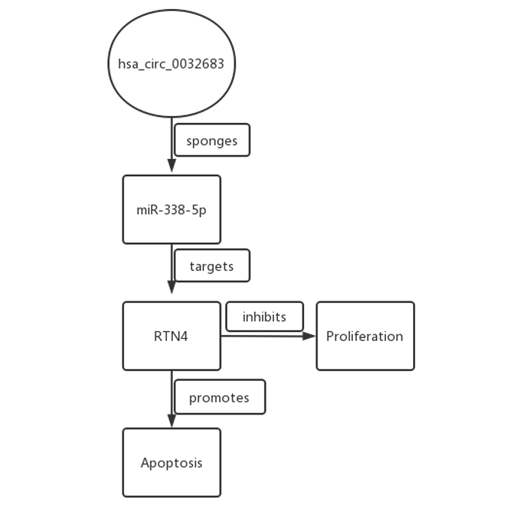

Supplement: Supplemental Material [file KBIE_A_2024961_SM8694.zip › supplementary/GraphicalAbstract.tif]
